# Case report: Kimura's disease with minimal degenerative glomerulopathy without eosinophil infiltration responds to mycophenolate mofetil treatment

**DOI:** 10.3389/fmed.2022.1069553

**Published:** 2023-01-09

**Authors:** Qing Han, Jie Han, Weitao Wang, Jie Gao, Youzhen Qiao, Junfeng Jia, Kui Zhang, Zhaohui Zheng, Ping Zhu

**Affiliations:** ^1^Department of Clinical Immunology, Xijing Hospital, Fourth Military Medical University, Xi'an, Shaanxi, China; ^2^National Translational Science Center for Molecular Medicine, Xi'an, Shaanxi, China; ^3^Department of Cardiovascular Surgery, Xijing Hospital, Fourth Military Medical University, Xi'an, Shaanxi, China

**Keywords:** mycophenolate mofetil, minimal degenerative glomerulopathy, eosinophil infiltration, Kimura's disease, treatment

## Abstract

Kimura's disease (KD) is a rare chronic progressive immune inflammatory disease. The etiology is unknown and manifests as a chronic inflammatory process, which is more common in young Asian men. The clinical manifestations are painless subcutaneous swelling of the head and neck and periauricular lymphadenopathy, which is slow growing and has a benign course. KD may involve the kidney, and pathological examination revealed eosinophil infiltration in the renal tissue. Proteinuria has been reported in 12–16% of KD cases, and about 60–70% of KD patients will develop nephrotic proteinuria. KD is easily confused with nephrotic syndrome, because KD does not have specific clinical manifestations, laboratory and imaging, and early misdiagnosis is easy. We report a case of KD that was biopsy-proven to have minimal lesion glomerulopathy after ~11 years. In this report, we describe a clinical case of KD with nephrotic syndrome, but there's no eosinophil infiltration in the kidneys. The clinical manifestations of KD recurrence were bilateral eyelid edema, bilateral lower limb swelling, and massive proteinuria in response to mycophenolate mofetil treatment (1.5 g).

## Introduction

Kimura disease (KD), also known as eosinophilic lymphatic granuloma, is a rare chronic inflammatory granuloma mainly characterized by damage to lymph nodes, soft tissues and salivary glands. Its etiology is unknown, and it mostly occurs in the subcutaneous soft tissues of the head and neck, mainly involving major salivary glands. Partial cases are prone to recurrence after local resection (1–3). KD is a benign chronic vascular lymphoid hyperplasia, the etiology of which remains unclear. It was associated with increased number of eosinophils and mast cells in peripheral blood and diseased tissues and increased levels of immunoglobulin (Ig) E, tumor necrosis factor (TNF) -α, interleukin (IL-4), IL-5, and IL-13 in serum. It is typically manifested as head and neck mass and can involve subcutaneous tissues, major salivary glands and lymph nodes (4, 5). We report a case of KD with a biopsie-confirmed course of microdegenerative glomerulopathy at 11 years of age. At year 11, the patient responded to treatment with mycophenolate Mofetil (1.5 g).

## Case report

**Figure 1:**
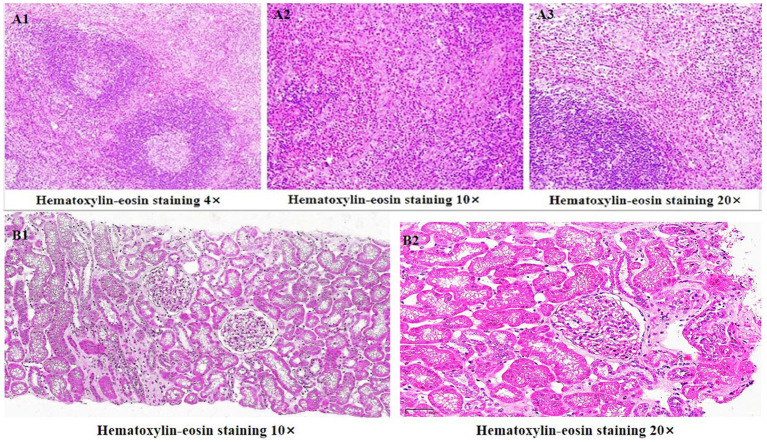
**(A1–A3)** Histological examination of lymph node biopsy: Reactive proliferation of lymphoid tissue in the neck behind the right ear, accompanied by follicular hyperplasia, epithelioid vascular endothelial hyperplasia and a large number of eosinophils infiltration. It's consistent with Kimura's degenerative lymphadenopathy. **(B1, B2)** Hematoxylin eosin staining showed no eosinophil infiltration in the kidney.

**Figure 2:**
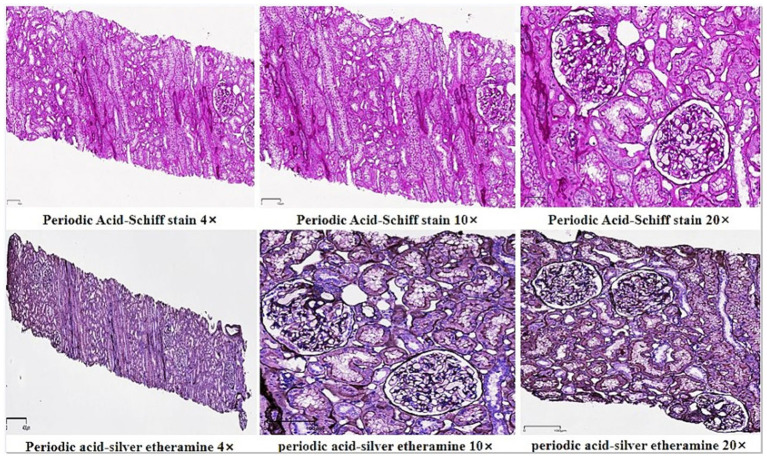
Mild segmental hyperplasia of mesangial cells and stroma. Vacuolar degeneration of basement membrane. There was mild focal atrophy of the renal tubules, occasional protein, red blood cell tube. Mild small focal fibrosis of interstitium, a small amount of chronic inflammatory cells and foam cell leaching Embellish. The arterioles were thickened. Consider minimal degenerative glomerulopathy.

In June 2011, an 11-year-old Chinese male patient presented with cystic swelling of 3 ×3 cm in the posterior and neck after upper respiratory illness, edema of both eyelids and lower limbs. Urinary protein showed 3+, and urinary protein quantification was 2741 mg/24h. Pathological biopsy in October 2011 suggested: Reactive hyperplasia of lymphoid tissue in the neck behind the right ear, accompanied by follicular hyperplasia, epithelioid vascular endothelial hyperplasia and numerous eosinophilia infiltration, was consistent with KD lymphadenopathy (Figure 1A). Hematoxylin eosin staining showed no eosinophil infiltration in the kidney (Figure 1B). The symptoms were relieved after administration of “prednisone 60 mg/d and tripterygium wilfordii polyside tablets 15 mg 2/d” according to “KD with nephrotic syndrome”. In December 2012, bilateral eyelid edema recurred after upper respiratory illness, accompanied by vomiting, which was stomach contents. The corticosteroid was adjusted to “prednisone 50 mg, qod stop tripterygium wilfordii”, and then the corticosteroid was reduced by 2.5 mg every week. In May 2013, the corticosteroid was stopped, and in June 2013, edema of lower extremities occurred without inducement, and the corticosteroid was adjusted to “prednisone 20 mg, qod”. In July 2013, urinary protein 2+ was adjusted to “prednisone 60 mg/d”, and urinary protein turned negative after reexamination. In November 2013, after a upper respiratory illness, urine protein 3+ was checked, and the treatment was adjusted to “prednisone 60 mg/day, anti-infection and diuretic treatment”, and the urine protein turned negative. In January 2014, edema recurred in both eyelids and lower limbs after a upper respiratory illness. He was treated with methylprednisolone sodium succinate 600 mg× 3 d High-dose corticosteroid therapy, and the condition improved. In February 2014, the above symptoms recurred, and renal puncture biopsy suggested minimal lesion nephropathy, so the patient was given “methylprednisolone sodium succinate 600 mg× 3 d High-dose corticosteroid therapy” again, followed by “prednisone 40 mg/d”. After that, the patient relapsed every time after upper respiratory illness, and the corticosteroid was adjusted to “30–50 mg/d” accordingly, and the condition was unstable. In June 2022, the above symptoms were repeated, 24 h urine protein quantification was 6.3 g, and erythrocyte sedimentation rate was 29 mm/h. Total IgE, immunoglobulin series (such as IgA), blood routine, coagulation series, autoantibody series, renal function, complement levels (C3, C4) were normal, and tests for Indicators of infection (e.g., HIV, hepatitis B surface antigen, hepatitis C virus) were negative. Renal puncture and biopsy indicated minimal glomerulopathy (Figure 2). Acne-like rash was observed on the face, swelling of both eyelids, swelling of both lower limbs, and a 3 × 3 cm swollen lymph node was palpable behind the left ear. Within 2 weeks of treatment with prednisone 40 mg and mycophenolate mofetil 1.5 g/d, the swelling decreased from 4 × 3 to 1 × 1 cm, facial edema and lower limb edema disappeared. The eosinophil count, urinary protein quantition, renal function and serum total IgE were all normal and no obvious side effects were observed. No new swelling was observed at 3 months of follow-up after treatment.

## Discussion

KD is often characterized by painless subcutaneous nodules or sclerosis, which mostly appear in the head and neck and can involve the internal organs. In the early stage, it is often misdiagnosed and missed because of no symptoms. KD has the possibility of multiple organ involvement. In previous reports, about 12% to 16% of Kimura's patients were complicated with nephropathy, including micropathological nephropathy, membranous nephropathy, IgA nephropathy, etc. In addition, those with diabetes, hypertension and cardiovascular and cerebrovascular diseases were 13.6, 23.7, and 8.5%, respectively. Rarely, acute mesenteric ischemia is associated with multiple small vessel embolism. The pathology is characterized by benign vascular lymph node hyperplasia accompanied by eosinophil infiltration. Lymph nodes (such as supratrochlear lymph nodes, axillary lymph nodes, and inguinal lymph nodes) and salivary glands (such as submandibular lymph nodes) are commonly involved, and may involve oral cavity, orbit, and sinuses (6–8). The lesion is usually a single or multiple enlarged lymph nodes with pruritus and is usually absent and prone to recurrence. The diagnosis is usually confirmed by pathological biopsy. Verruca-finkeldi type multinucleated cells can be seen (9, 10). Lymph nodes were damaged in > 90% of KD patients. Microscopic examination in such patients showed the interfollicular region and capsule of lymph nodes. In some patients, there was a large amount of eosinophilic infiltration in the epidural adipose tissue, which could form eosinophilic microabscess (11, 12). The degree of eosinophilia may be related to lymph node size. The pathogenesis of KD has not been determined, but there is evidence that dysregulation of T-cell immunity plays a pathogenic role (7). Nephrotic syndrome can be associated with KD in Asia, which is also a very rare case report and has been successfully treated with mycophenolate mofetil. Eosinophil infiltration was reported to be uncommon in renal tissue of patients with KD involvement, suggesting that renal injury may not be caused only by eosinophil infiltration directly, but may also be caused by other factors (such as T-cell regulation is disturbed) (11–17). At present, there is no unified standard for the diagnosis of KD combined with kidney involvement, and the clinical diagnosis is mainly based on the diagnosis of KD and the clinical manifestations of kidney damage. The diagnosis of KD is mainly based on clinical and histopathological features. In recent years, some literatures have reported that the imaging manifestations of KD in the head and neck have certain characteristics which can assist the diagnosis. KD needs to be distinguished from a variety of benign diseases, there is no exact treatment plan, but also need to exclude other malignant tumor diseases with similar clinical manifestations (14, 15). The recurrence of the disease is affected by many factors, such as the length of the disease, the young age of onset, the size of lymph nodes, the proportion of eosinophils, the serum IgE level, and the extent of the lesion (18).

We report the patient's proteinuria responded well to mycophenolate mofetil at 1.5 g/d and had no recurrence of proteinuria during the 3-month after treatment. KD is currently considered to be a type I allergic reaction disease mediated by IgE mediated by abnormal regulation of CD4^+^T helper cells and a high possibility of autoimmune disease. The number of eosinophils increased in peripheral blood and diseased tissues, and the levels of serum inflammatory factors such as IgE, TNF-α, IL-4, IL-5, and IL-13 increased. This case reported that mycophenolic acid may have positive therapeutic effects on KD with nephrotic syndrome with massive proteinuria, eyelid edema and lower limb swelling. Mercaptopropionic acid (MPA) can inhibit the proliferation of human T lymphocytes to mitosis. Inhibit the proliferation of human B lymphocytes to T-cell – dependent and T-cell – independent antigens. MPA also has an impact on the interaction between leukocyte and endothelial cells, which can reduce the adhesion between T cells and endothelial cells and cannot penetrate the endothelium to reach the inflammatory area, so it has potential infiltration blocking activity. In animal models, MPA significantly inhibited the expression of Th2 cytokines, but did not affect the expression of Th1 cytokines. However, long-term and clinical follow-up and observational studies are needed to determine the mechanism of its long-term efficacy, safety and efficacy.

The diagnosis of KD is mainly based on clinically painless subcutaneous masses of the head and neck and local lymph node enlargement, especially in patients with peripheral blood eosinophils and elevated serum lgE. The possibility of KD should be considered, and further tumor or lymph node biopsy should be performed to confirm the diagnosis. However, the cases reported here lack some typical clinical and laboratory features. Diagnosis is mainly by histological examination. In the future, more typical cases need to be accumulated and their therapeutic effects tracked. It was found that peripheral blood eosinophil percentage ≥ 20%, maximum mass diameter, skin pruritus, and history of recurrence after previous treatment were positively correlated with disease progression. The percentage of peripheral blood eosinophil ≥ 20% is an independent risk factor for the prognosis of KD. In the past, KD was considered as a chronic benign disease with no tendency to become malignant. In recent years, several cases of renal failure in patients with nephrotic syndrome and lymphoma in patients with KD have been reported. It is suggested that KD is not only easy to relapse, but also may lead to serious cases. Follow-up of KD should be strengthened.

## Conclusion

In conclusion, KD should be cautioned in patients with heavy proteinuria, especially in adolescent males with painless subcutaneous soft tissue masses associated with increased peripheral eosinophils, double-stranded DNA negative, and elevated serum IgE. KD pathology is characterized by benign vascular lymph node hyperplasia accompanied by eosinophil infiltration. Early pathological biopsy (such as kidney biopsy and lymph node biopsy) is helpful for early diagnosis and guidance of treatment. At present, the pathogenesis of KD involvement in the kidney has not been fully elucidated. Due to the lack of understanding of the disease, it is easy to be misdiagnosed clinically, and diagnosis and treatment need to be unified. Therefore, further clinical and basic research is needed in the future.

## Data availability statement

The data that support the findings of this study are available from the corresponding author, upon reasonable request.

## Author contributions

QH and JH: wrote, collected data, and design. WW, JG, and YQ: collect data. JJ and KZ: design. ZZ and PZ: design, supervise, and direct. All authors contributed to the article and approved the submitted version.
